# Supplementary figures and images for: Precursor RNA structural patterns at SF3B1 mutation sensitive cryptic 3’ splice sites
Source: bioRxiv. 2025 Feb 22:2025.02.19.638873. Preprint. [Version 1] doi: 10.1101/2025.02.19.638873 (PMC11870503; doi:10.1101/2025.02.19.638873)

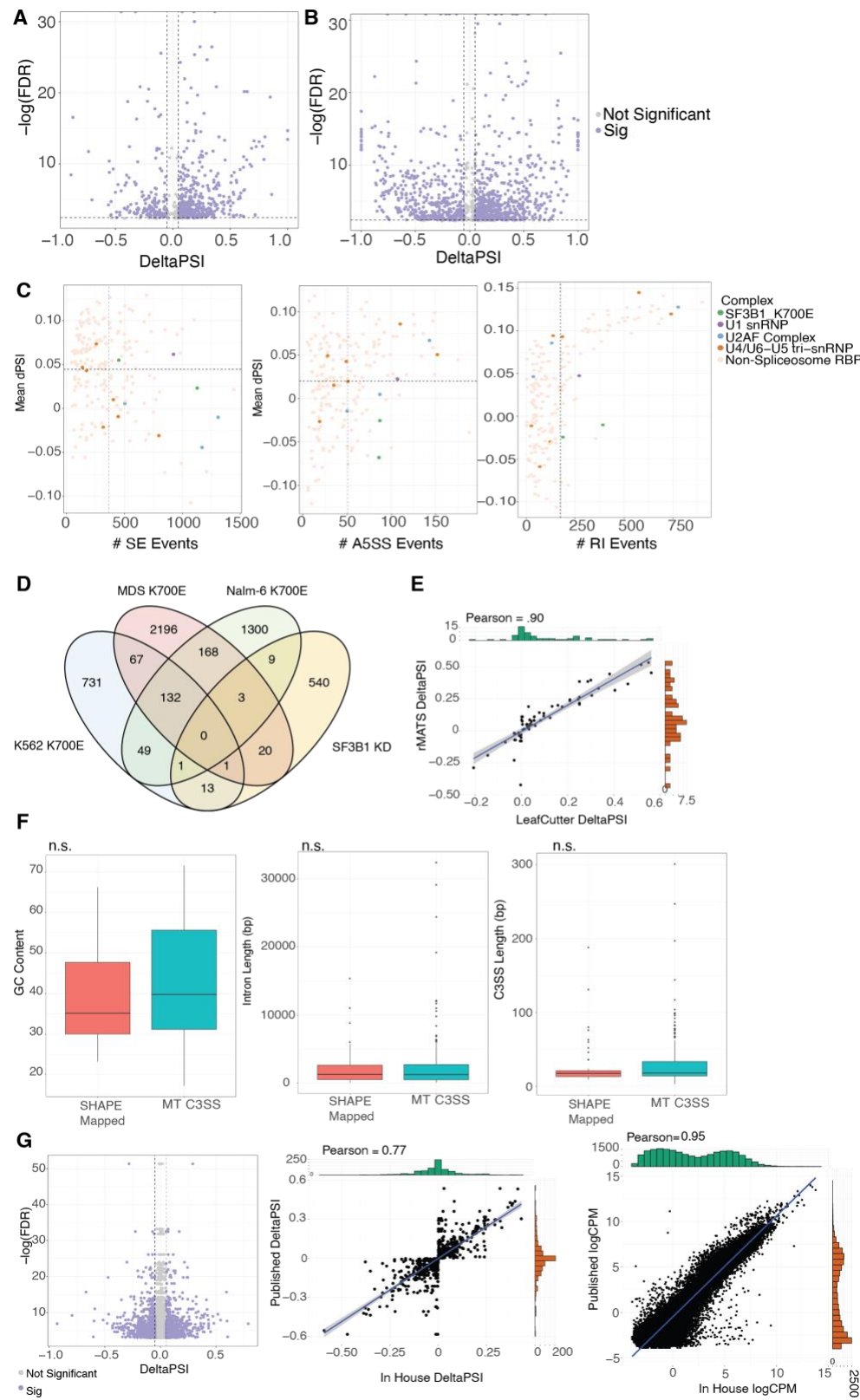

**Supplementary Figure 1**

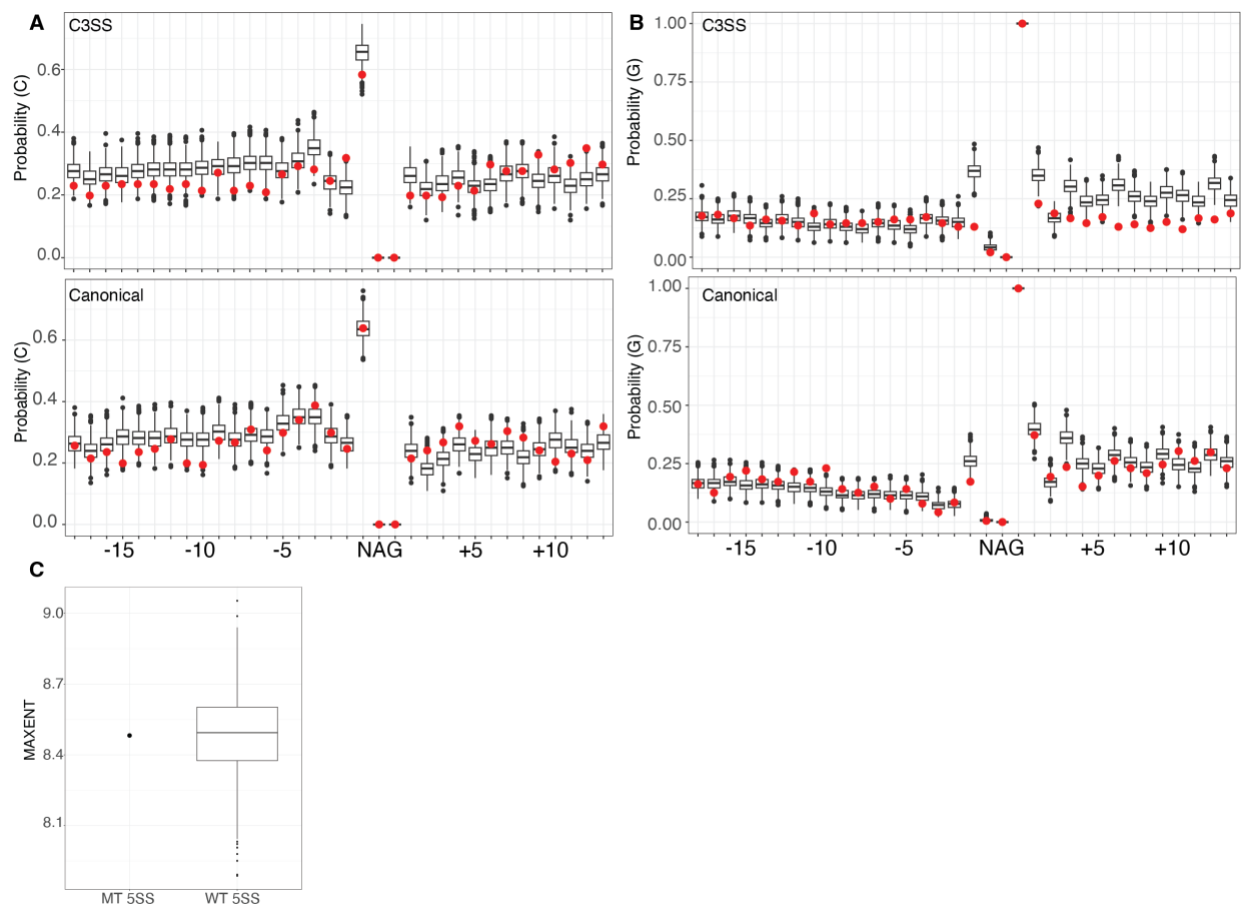

Supplementary Figure 2

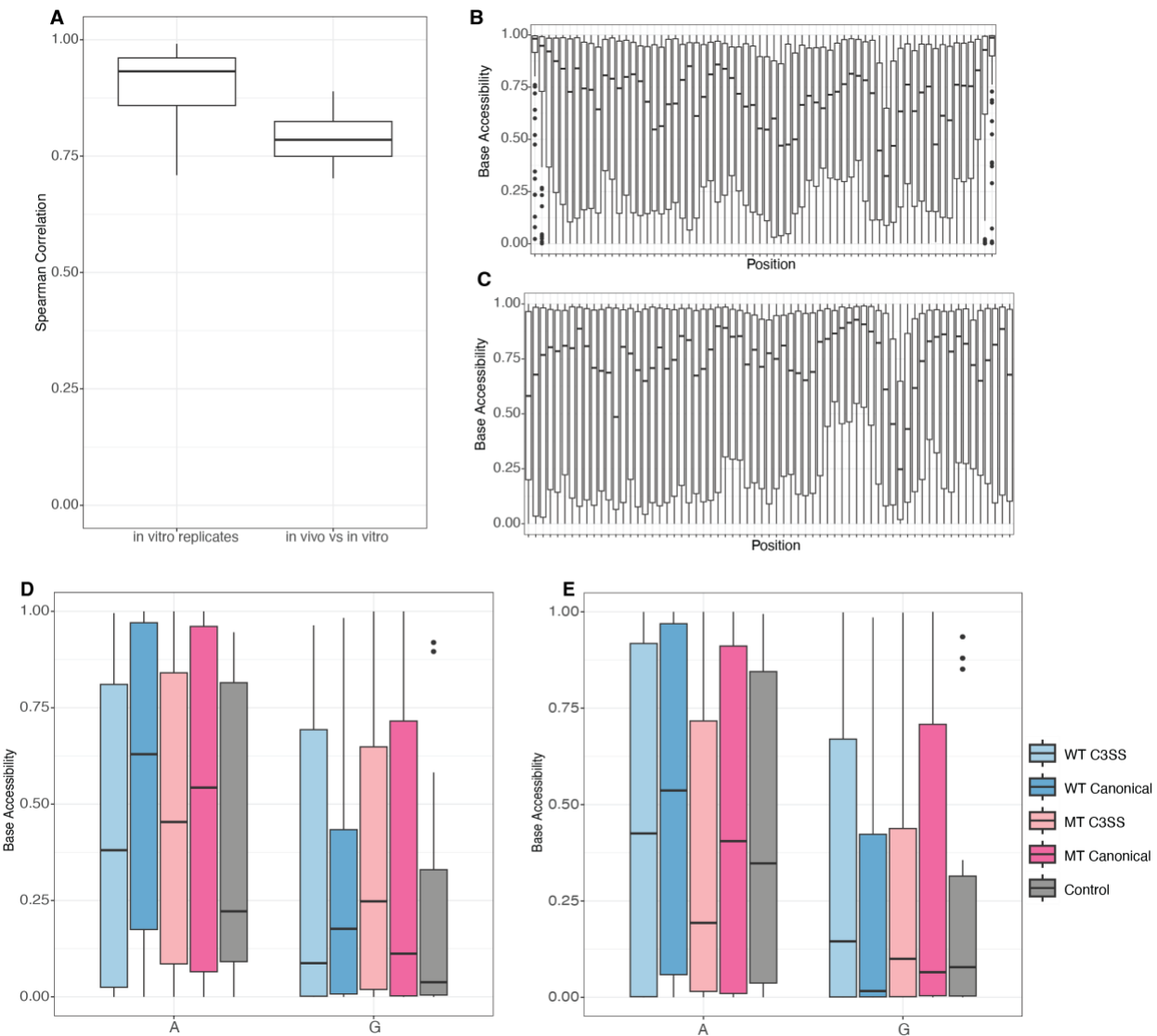

Supplementary Figure 3

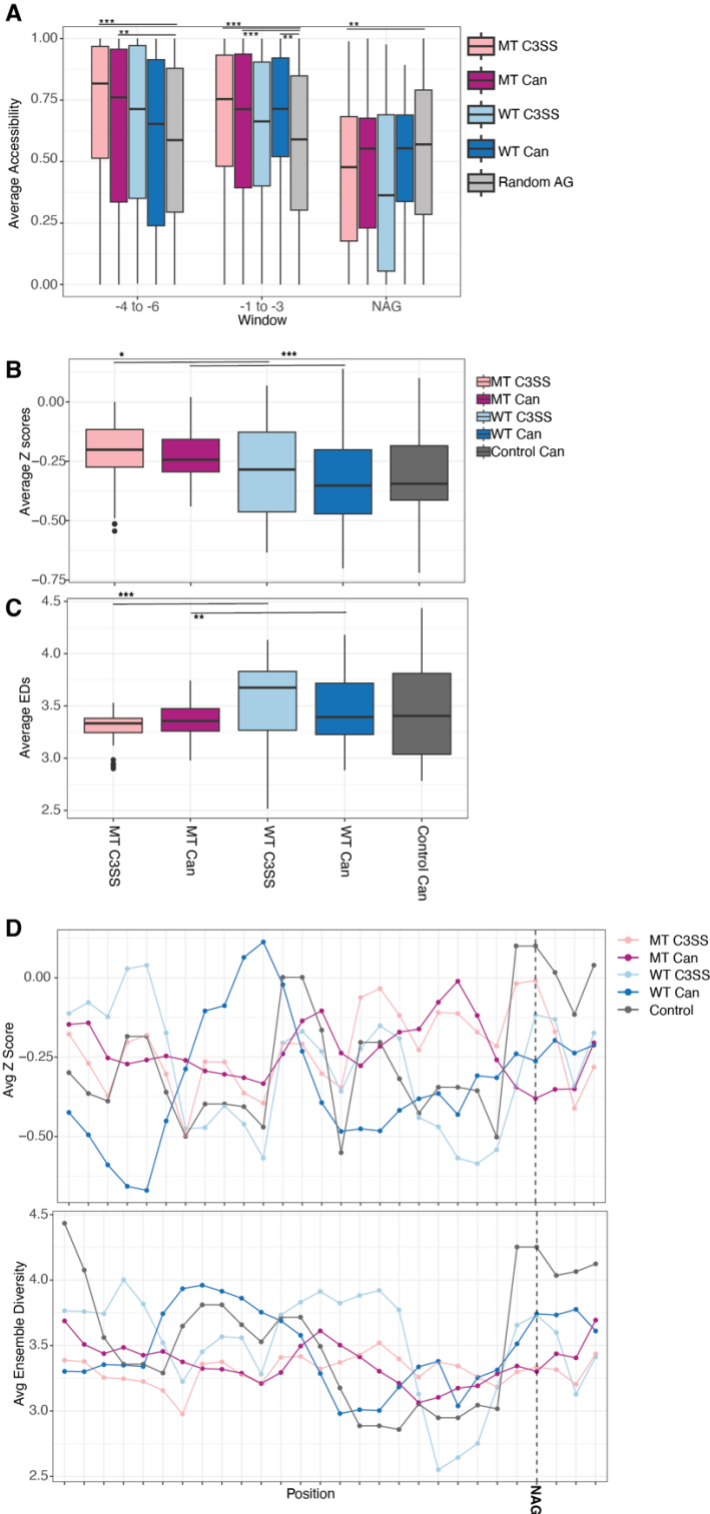

Supplementary Figure 4

Supplement: 1 — Supplementary Figure 1. SF3B1 K700E mutants display unique splicing patterns. (A) SF3B1 knockdown in HepG2 (ENCSR896CFV) cells exhibit many alternative splicing events, FDR < .05, deltaPSI > .05. (B) SF3B1 K700E mutation in K562 cells display many alternative splicing events. (C) Little to no trend is observed in exon skipping, alternative 5’ splicing and intron retention events in SF3B1 K700E mutants. Number of and percent spliced in of skipped exons, alternative 5’ splice sites, and intron retention events across ENCODE RBP knockdown series and SF3B1 K700E cell lines. (D) SF3B1 K700E mutants and SF3B1 knockdowns share little overlap in alternative splicing events. Overlap in rMATS C3SS events between SF3B1 K700E cell types and SF3B1 knockdown in HepG2 cells. (E) Alternative splicing analysis with rMATs and LeafCutter display a high correlation. Correlation of deltaPSI in C3SS events called from rMATS and LeafCutter in SF3B1 K700E Nalm-6 cells. (F) Cryptic 3’ splice site sensitive to SF3B1 mutation chosen for structure mapping (n=83) are statistically similar in GC content, intron length, and C3SS length to the entire pool (n=192). (G) In-house sequenced NALM-6 SF3B1 K700E display high correlation in gene expression and alternative splicing profiles with previously published data. Supplementary Figure 2. SF3B1 MT C3SS and paired canonical splice sites have similar GC nucleotide composition. (A) SF3B1 MT cryptic 3 splice sites (MT C3SS) and paired canonical display similar probabilities for cytosine and (B) guanine in MT C3SS (top) and paired canonical (bottom) plotted versus randomly bootstrapped WT C3SS and paired canonical. (C) No significant different in the MAXENT of 5’ splice sites paired to SF3B1 MT C3SS and WT C3SS. Supplementary Figure 3. SHAPE correlation and SHAPE guided accessibility patterns. (A) A high spearman correlation is observed between in vitro replicates (n=83) and in vitro versus in vivo structure mapped introns (n=5) > .7. (B) Folding short s [file NIHPP2025.02.19.638873V1-supplement-1.pdf]
